# What could prevent chronic condition admissions assessed as preventable in rural and metropolitan contexts? An analysis of clinicians’ perspectives from the DaPPHne study

**DOI:** 10.1371/journal.pone.0244313

**Published:** 2021-01-07

**Authors:** Jo Longman, Jennifer Johnston, Dan Ewald, Adrian Gilliland, Michael Burke, Tabeth Mutonga, Megan Passey

**Affiliations:** 1 University Centre for Rural Health, The University of Sydney, Lismore, NSW, Australia; 2 North Coast Primary Health Network, Ballina, NSW, Australia; 3 Coffs Medical Centre, Coffs Harbour, NSW, Australia; 4 School of Medicine, The University of Sydney, Sydney, NSW, Australia; 5 School of Medicine, Western Sydney University, Sydney, NSW, Australia; 6 Mt Druitt Community Health Centre, Western Sydney Local Health District, Mt Druitt, NSW, Australia; Waikato Institute of Technology, NEW ZEALAND

## Abstract

**Introduction:**

Reducing potentially preventable hospitalisations (PPH) is a priority for health services. This paper describes the factors that clinicians perceived contributed to preventable admissions for angina, diabetes, congestive heart failure (CHF) and chronic obstructive pulmonary disease (COPD), and what they considered might have been done in the three months leading up to an admission to prevent it.

**Methods:**

The study was conducted in a rural and a metropolitan health district in NSW, Australia. Expert Panels reviewed detailed case reports to assess preventability. For those admissions identified as preventable, comments from clinicians indicating what they perceived could have made a difference and/or been done differently to prevent each of the preventable admissions were analysed qualitatively.

**Results:**

148 (46%) of 323 admissions were assessed as preventable. Across the two districts, the most commonly identified groups of contributing factors to preventable admissions were: ‘Systems issues: Community based services missing or inadequate or not referred to’; ‘Patient issues: Problems with adherence/self-management’; and ‘Clinician issues: GP care inadequate’. In some instances, important differences drove these groups of factors. For example, in the rural district ‘Systems issues: Community based services missing or inadequate or not referred to’ was largely driven by social and welfare support services missing/inadequate/not referred to, whereas in the metropolitan district it was largely driven by community nursing, allied health, care coordination or integrated care services missing/inadequate/not referred to. Analyses revealed the complexity of system, clinician and patient factors contributing to each admission. Admissions for COPD (rural) and CHF (metropolitan) admissions showed greatest complexity.

**Discussion and conclusion:**

These findings suggest preventability of individual admissions is complex and context specific. There is no single, simple solution likely to reduce PPH. Rather, an approach addressing multiple factors is required. This need for comprehensiveness may explain why many programs seeking to reduce PPH have been unsuccessful.

## Introduction

Potentially preventable hospitalisations (PPH) are unplanned admissions considered to be potentially preventable with appropriate outpatient care prior to the admission, though there is no standard time frame. The concept was first described in America three decades ago and is considered a proxy measure of the effectiveness of primary healthcare. In Australia, the rate of PPH is tied directly to health service funding [1, 2]. Admissions for chronic conditions (almost entirely for congestive heart failure (CHF), chronic obstructive pulmonary disease (COPD), diabetes complications and angina pectoris) make up approximately half of all Australian PPH [3].

Reducing PPH is a priority for health services. Whilst the rate of PPH for chronic conditions (excluding diabetes) has declined in Australia between 2013–14 and 2017–18 [4] there remain significant costs for the healthcare system, the hospital, clinicians, patients and their carers/families for every hospital admission, and admissions which are deemed potentially preventable are an obvious target for action. The aim of the DaPPHne (Diagnosing Potentially Preventable Hospitalisations) study was to better understand and therefore contribute to the design of interventions to further reduce PPH for chronic conditions.

Chronic PPH are defined by an agreed list of discharge codes, for example an admission with a primary diagnosis on discharge of a diabetes complication would be classified as a PPH. However, this definition captures all admissions for conditions on the list regardless of whether they were, in fact, preventable and thus overestimates preventable admissions for those conditions [5, 6] (it also has the potential to fail to identify preventable admissions for diagnostic codes which are not specified in the classification list).

The study described in this paper was a sub-study of the wider DaPPHne study [7, 8]. The DaPPHne study identified the proportion of admissions for CHF, COPD, diabetes complications and angina pectoris that were actually preventable and the factors which predicted the preventable admissions. This knowledge makes a significant contribution to targeting interventions, and the broad nature of those interventions, such as the need for social and welfare support.

In the current paper we add to this understanding by providing perspectives from clinicians involved in the DaPPHne study about what contributed to each PPH they assessed as preventable, and what could have made a difference in the three months leading up to the admission. This will help to develop the detail of interventions to reduce PPH.

## Methods

### The wider DaPPHne study

**Fig 1:**
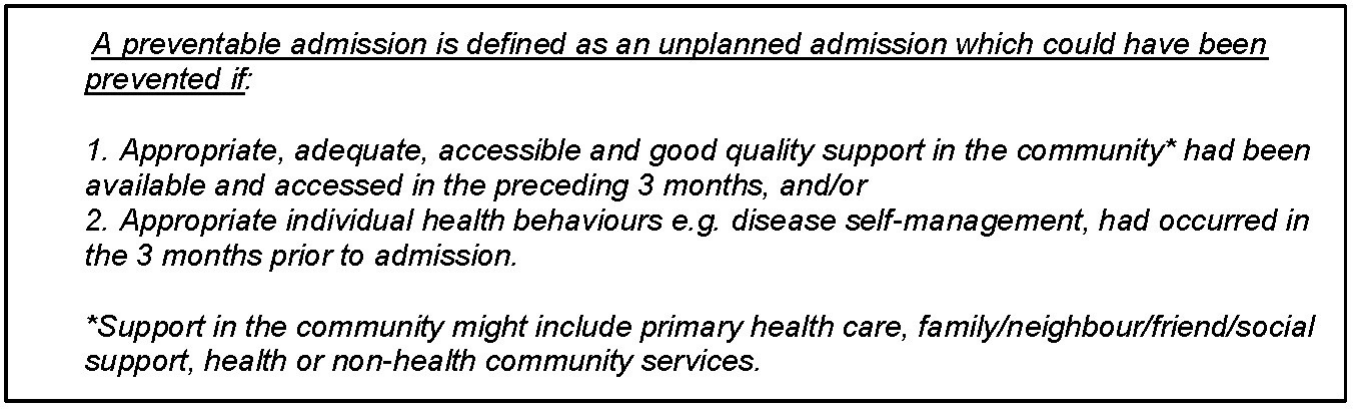
The definition of a potentially preventable hospitalisation.

In the wider DaPPHne study, patients with an admission for one of four chronic conditions (CHF, COPD, angina or diabetes) were recruited. For those admissions with complete data, an Expert Panel then comprehensively reviewed each admission to assess which ones were actually preventable [9], using the definition in Fig 1 below.

This definition makes the assumption that all individual health behaviours are modifiable, as it was agreed that the Panel would not necessarily be able to make a clear assessment of which health behaviours could have been changed [9]. A three-month time frame was chosen to focus on secondary prevention of relapses and hospitalisations, and was deemed most likely to guide short to medium-term interventions rather than primary prevention of underlying conditions.

**Fig 2:**
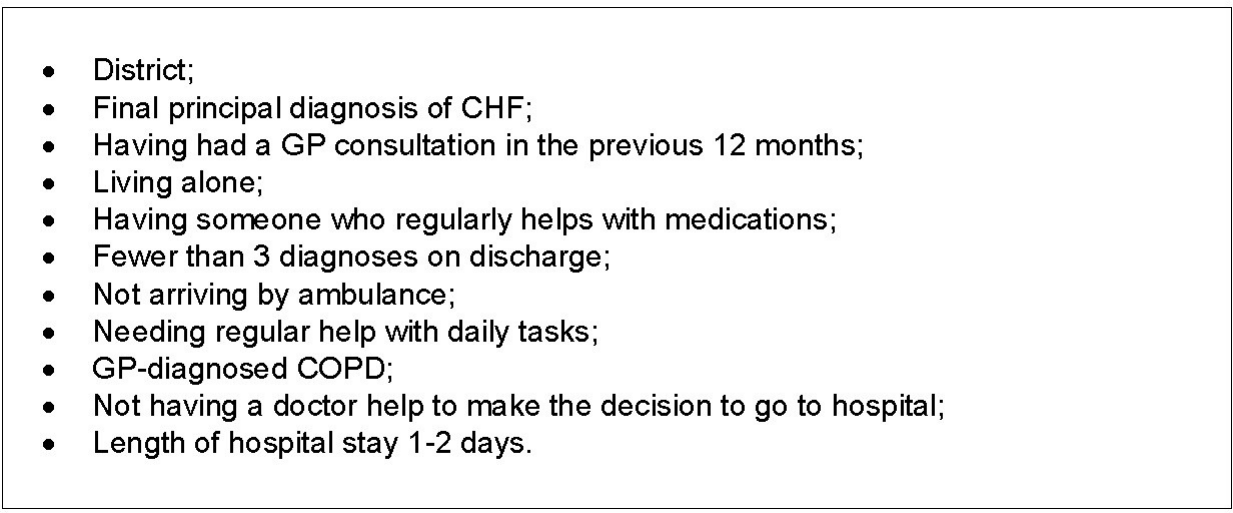
Predictors of admissions classified as preventable in the DaPPHne study.

The Panels’ assessments of preventability identified a group of admissions that were preventable. These were compared to a second group consisting of the remaining admissions assessed as NOT preventable and those where it was not possible to categorise the admission as either preventable or not preventable. Thus the factors that predicted a preventable admission (from a multivariate model adjusted for age, sex and Indigenous status) were discerned [8] (Fig 2).

The wider DaPPHne study methods have been described elsewhere [7]. The study was conducted in two rural hospitals (total of 487 beds) and one metropolitan hospital (570 beds) from two health districts of NSW, Australia. Research nurses recruited patients and collected data. Research nurses received two days of training in research methods, the study protocol and procedures and received ongoing supervision by the research team. Participants were patients with an admission for one of the four chronic conditions, aged 45 years or over, community-dwelling, able to give informed consent and not transferred from another hospital. In the rural district, data were collected between October 2014 and March 2016, and in the metropolitan district between January 2016 and June 2017. An Expert Panel of three clinicians was established for each of the three DaPPHne hospitals and reviewed detailed case reports of each admission to assess the preventability of that admission, following an enhanced version [9] of the approach taken by Oddone [10] and Arozullah [11].

Each Expert Panel consisted of a general practitioner (GP), chronic disease nurse and a specialist physician, all of whom worked in the local area for that hospital and had detailed knowledge of the healthcare systems and health services available. The case reports contained information from: an interview with the patient, an interview with the patient’s GP, an audit of the hospital notes, the hospital clinical notes from the first 24 hours of the admission and the discharge summary. The clinicians on each Panel therefore had access to comprehensive information for every admission, allowing for an in-depth consideration of the factors which may have come into play in the three months leading up to the admission.

The Panel members, who all attended training in the assessment of preventability (three hours of training and practising assessments followed by discussion) and received ongoing support from the research team, were asked to independently assess if they were *reasonably confident* this admission was preventable using the definition of a preventable admission (Fig 1). The other two assessment options were that they were *reasonably confident* the admission was not preventable or that they were unable to categorise the admission as preventable or not. For those admissions where there was not agreement between the panel members, a facilitated discussion was convened where consensus was reached.

### The sub-study described in this paper

For those admissions assessed as preventable, the Panel members were asked to indicate what could have made a difference and/or been done differently to prevent that admission, given currently available services. They were also asked to suggest any improved or additional services or social support which, if available, could have helped prevent the admission. Panel members wrote brief, free-text (i.e. unstructured, written) responses to these questions. Similarly, structured interviews were conducted by the research nurses with patients’ GPs, face-to-face or telephone, and included GPs commenting briefly on what could have made a difference and/or been done differently to prevent the admission (given currently available services or any improved or additional services or social support).

Participants in this sub-study were therefore clinicians on the Expert Panel for each hospital and GPs of patients from the DaPPHne study. Comments from all these clinicians were the data that were qualitatively analysed for this paper. The proportion of cases coded to particular groups of contributing factors and how the contributing factor groups are ranked in rural and metropolitan districts are also provided to aid interpretation.

### Qualitative (free-text) data analysis

This sub-study of the DaPPHne study was methodologically underpinned by pragmatism, aiming to ask specific questions with a focus on the utility of the answers [12]. Coding took place in two phases using content analysis following Elo [13]. Data for the rural district were available first. The free-text data from the Panels and GP interviews were read and reread and deductively coded largely using *a priori* codes which were developed based on the literature and our previous research [14–17]. In addition, a small number of new codes were developed or existing codes nuanced when the coding frame did not adequately fit the content of the free-text. The final coding frame was developed (by JL and MP–a social scientist and an epidemiologist/public health researcher both with considerable experience in qualitative research), based on analysis of free-text data from six admissions deemed preventable (selected with the aim of maximising variation in condition, patient characteristics and amount and richness of free-text data). These six were double-coded to check consistency of coding and interpretation, with any lack of consistency discussed and resolved [13]. After this, the coding frame was fixed and all the free-text comments were coded using it. Data were coded to multiple codes.

Free-text data for the metropolitan district were available later. Coding (by JL and JJ a psychologist experienced in qualitative research) began with six of the admissions deemed preventable using the final coding frame used for the rural district data, to check the ‘fit’, and minor amendments made. The amendments primarily related to the specific services clinicians felt patients might have been referred to which may have prevented the admission, which differed substantially between the two health districts. The same process was adopted as for the rural district data in developing a final coding frame that all the metropolitan district preventable admission free-text data were subsequently coded to.

Throughout the coding, an ‘additional detail’ journal was kept containing specific examples illustrating the codes. The items in the journal were discussed at length and added depth of understanding to the analyses.

Individual codes in each of the two datasets (one rural one metropolitan) were organised into ‘contributing factor groups’ where content was similar, under three broad categories–patient, clinician or system factors [7]. These groups are shown in Tables 3 and 4, with a description of the codes included in each group.

### Ethics

The study was approved by the NSW Population and Health Services Research Ethics Committee (AU RED Reference: HREC/14/CIPHS/39, Cancer Institute NSW reference: 2014/06/538). All patients participating in the study provided written informed consent prior to any study-related data being collected.

## Results

### Wider DaPPHne study sample

In total, 7,822 admissions were screened, 1,808 were potentially eligible for the study and patients for 791 admissions consented to participate in the DaPPHne study, with 240 subsequently excluded as the final diagnoses were not consistent with the DaPPHne inclusion criteria [8]. Six patients withdrew. This left 545 eligible admissions. Of these 545 admissions, data from all data sources (‘complete’ cases) were collected for 323 (in all incomplete cases, an interview with the patient’s GP was missing), with these 323 admissions subsequently assessed by their local Expert Panel. Admissions missing data from a GP interview were not assessed by the Panel as it was agreed that this was vital information without which it was too difficult to assess preventability. Differences between admissions assessed and not assessed by the panels existed. Those assessed by the panels had better health literacy, lower psychological distress and were less likely to have seven or more diagnoses on admission, indicating they may be slightly healthier than those not assessed by the panel. Further detail on the differences is available in the S1 File.

**Fig 3:**
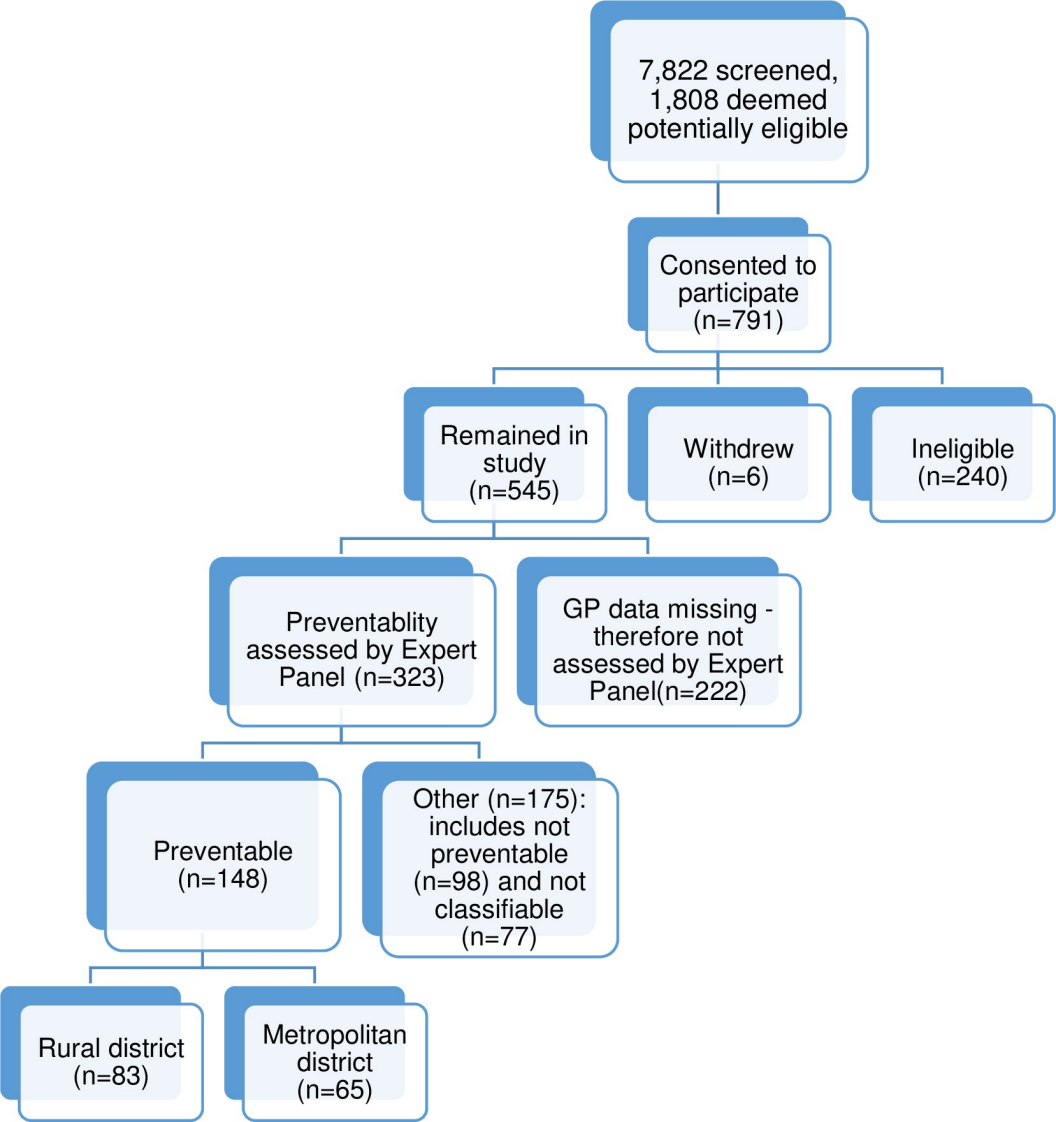
Admissions in the DaPPHne study: Numbers assessed and deemed preventable.

The flow of admissions through the DaPPHne study is described in Fig 3 below.

### Preventable admissions

Of the 323 case reports reviewed, the Panels deemed 148 to be preventable. The proportion preventable varied significantly by district and by final principal discharge diagnosis with the majority of admissions for CHF that were assessed being classified as preventable (Table 1). Further detail on this aspect of the study is covered in a separate paper [8].

**Table 1:** Panel assessment of preventability by hospital and final principal discharge diagnosis.

	Preventable n = 148 (%)	Not preventable or not classifiable n = 175 (%)	P value
**Panel assessment by district**			
Rural district	83 (39)	127 (61)	
Metropolitan district	65 (58)	48 (42)	
			*<0*.*001*
**Panel assessment by final principal discharge diagnosis**			
CHF	50 (63)	29 (37)	
COPD	58 (47)	65 (53)	
Angina	9 (27)	24 (73)	
Diabetes	31 (35)	57 (65)	
			*<0*.*001*

This paper presents findings for the 148 admissions assessed as preventable. The demographic characteristics of the admissions assessed as preventable by the Expert Panels are shown in Table 2. This table highlights differences in the two districts, for example the patients with preventable admissions from the metropolitan district were more likely to have been born in countries other than Australia, and have higher levels of education, health literacy and to have seen their GP about their condition in the three months prior to admission.

**Table 2:** Demographic characteristics of admissions assessed as preventable by the Expert Panels (n = 148)–rural compared to metropolitan district.

	Rural N = 83 n (%)	Metro N = 65 n (%)	P-value
***Demographics***			
**Gender**			0.075
Male	48 (58)	28 (43)	
Female	35 (42)	37 (57)	
**Age**			0.384
46–60 years	13 (15)	14 (22)	
60–70 years	23 (28)	22 (34)	
70–80 years	28 (34)	14 (22)	
80 and above	19 (23)	15 (23)	
**Country of Birth**			0.001
Other countries	16 (19)	29 (45)	
Australia	67 (81)	36 (55)	
**Aboriginal/Torres Strait Islander**			0.509
Indigenous	6 (7)	3 (5)	
Non-Indigenous	77 (93)	62 (95)	
**Relationship Status**			0.118
Widowed/Divorced/Single	43 (52)	42 (65)	
Married/De facto	40 (48)	23 (35)	
**Living Alone**			0.350
Lives Alone	30 (36)	28 (44)	
Other	53 (64)	36 (56)	
**Highest Level of Education**			0.039
No School certificate or equivalent	32 (39)	22 (34)	
School/Intermediate certificate	22 (27)	7 (11)	
High school certificate	3 (4)	7 (11)	
Trade/Apprenticeship	18 (22)	23 (35)	
University	7 (9)	6 (9)	
**Employment Status**			0.933
Retired	61 (73)	49 (75)	
Employed	6 (7)	5 (8)	
Others	16 (19)	11 (17)	
**Household Income (AUD)**			0.013
<$20,000	25 (31)	25 (58)	
$20,000-$40,000	41 (51)	12 (28)	
>$40,000	14 (18)	6 (14)	
**Insurance Status**			0.191
Others	63 (76)	55 (85)	
Private insurance	20 (24)	10 (15)	
***Self-reported health functioning***			
**Requires Daily Help**			0.203
Yes	33 (40)	33 (51)	
No	49 (60)	32 (49)	
***Health literacy***			
**Partners in Health scale**			0.006
Satisfactory/very poor	47 (57)	22 (34)	
Very good	36 (43)	43 (66)	
**REALM-R score**			0.503
Below 9^th^ grade	19 (23)	18 (28)	
9^th^ grade or higher	64 (77)	47 (72)	
***Self-reported social isolation*, *psychological distress***			
**Social support (Dukes)**			0.600
Little/No social support	20 (25)	13 (21)	
Moderate/High social support	61 (75)	49 (79)	
**Psychological distress—K10**			0.350
Moderate/High psychological distress	28 (34)	26 (41)	
Well/Mild psychological distress	55 (66)	37 (59)	
***Use of prescription medications***			
**Total medications on admission**			0.165
Five and above	61 (73)	54 (83)	
None to four	22 (27)	11 (17)	
**Someone helps with medications**			0.056
Yes	22 (27)	9 (14)	
No	59 (73)	55 (86)	
***Use of community-based services***			
**Saw GP about admission condition in previous 3 months**			0.009
Yes	56 (67)	56 (86)	
No	27 (33)	9 (14)	
***Diagnoses (as recorded in hospital records)***			
**Principal diagnosis on discharge**			0.048
CHF	30 (36)	20 (31)	
COPD	26 (31)	32 (49)	
Diabetes	23 (28)	8 (12)	
Angina/ACS	4 (5)	5 (8)	
**Total diagnoses on discharge**			0.112
1–2 conditions	11 (13)	3 (5)	
3–6 conditions	31 (37)	21 (32)	
7 and above	41 (49)	41 (63)	
***General practice management (GP Interview)***			
**Chronic conditions other than CHF, COPD, angina and diabetes**			0.011
Yes	77 (94)	52 (80)	
No	5 (6)	13 (20)	
**Social issues that impact on ability to manage their health**			0.913
Yes	51 (62)	41 (63)	
No	31 (38)	24 (37)	

### Analysing data from the two districts separately

Qualitative analyses were conducted separately for the rural and metropolitan data because the context for the study was somewhat different in each district. These differences included socio-demographic characteristics, for example in addition to the information in Table 2 above, the rural district population (for the whole population not just those aged 45 or over or those in the DaPPHne study) had predominantly Australian or English ancestry (around 60%), whereas the metropolitan district was more ethnically diverse (28% had Australian or English ancestry) and had lower proportions of the population speaking only English at home (44% in metropolitan vs 86% rural). The age structures of the populations were quite distinct with the rural population having a greater proportion of older people (around a quarter of the rural district population vs 12% of the metropolitan district were 65 or older). The population of the metropolitan district had a higher median weekly household income than the rural district population ($1,460 vs $1,045). In addition, the health support services for the kinds of patients in the DaPPHne study varied by district in terms of type, variety and availability as outlined above.

### Results of qualitative coding of data

Free-text data for the 83 and 65 admissions deemed preventable by the Expert Panels for the rural and metropolitan districts respectively, were initially coded to individual codes. These individual codes were then gathered into groups of contributing factors, for example missing or inadequate or not referred to allied health, social and welfare support, and rehabilitation services codes were gathered together under a group called ‘Systems issues: Community based services missing or inadequate or not referred to’. There were 15 contributing factor groups for the rural district and 14 for the Metropolitan district, under three broad categories–system issues (pink column), clinician issues (blue column) or patient issues (green column). The one group not required for the metropolitan hospital was ‘Clinician: Other’. This contained one code, ‘iatrogenesis’, which was rarely used, (n = 5) in the rural data.

The contributing factor groups and number of admissions coded to that group (each admission was coded to multiple groups) are shown in Tables 3 (rural district) and 4 (metropolitan district), including a description of the individual codes included in each group. Bold text indicates the name of the group, and underlined codes within that were the key drivers (in terms of most frequently used) within the group. The order in which the groups appear indicates the most common to least common within each category i.e. the first row contains the most commonly used groups within each of the three categories (Systems, Clinician and Patient).

**Table 3:** Rural district—coding of free-text comments for each of the 83 preventable admissions.

SYSTEMS Codes	CLINICIAN Codes	PATIENT codes
**Community based services missing or inadequate or not referred to (79 admissions coded to this group):**	**GP care inadequate (62 admissions coded to this group):**	**Problems with adherence/self-management (66 admissions coded to this group):**
	• inadequate medical management of existing chronic condition	
		• patient/carer requires education
e.g. social and welfare support, allied health, rehabilitation, Aged Care Assessment/needs assessment, community nursing, respite, etc.	• lack of action plan	• poor adherence to medication
		• inadequate self-management skills
	• inadequate GP medical management of known conditions predisposing to admission e.g. mental health, co-morbidities	
	• missing or inadequate GP Management Plan/Team Care Arrangements (Chronic Disease Management Plan)	
**Poor communication and linkages between services (43 admissions coded to this group):**	**Both GP and hospital (24 admissions coded to this group):**	**Problems with patient’s engagement with existing services (54 admissions coded to this group):**
	• failure to refer to existing community based health service e.g. palliative care, cardiac nurse	
		• declined services
• poor discharge practices between acute and primary care		• should have seen the GP earlier/sooner/more frequently
	• required home medicine review	
• between community based services		• has a GP but insufficient or inadequate connection with GP
		• should have gone to GP not the hospital
**Problems with specialist services (35 admissions coded to this group):**	**Hospital care (15 admissions coded to this group):**	**Support needed (34 admissions coded to this group):**
• unable to see specialist when required	• inadequate hospital management of existing condition	• lack of social support
		• support from existing carer inadequate/not coping at home
• needing access to or referral to specialist services locally	• specialist admitting unnecessarily for diagnostics that could have been done in the community	
		• support needed for carer/carer illness/death, and/or respite required
• publicly available cardiology services		
	• complication from a previous hospital admission	
**Other (28 admissions coded to this group):**	**Other (5 admissions coded to this group):**	**Cost/logistics barriers to accessing services (30 admissions coded to this group):**
• Could have been seen as outpatient or in the community	• iatrogenisis	
		• prohibitive costs of specialist services
• delay in access to diagnostics (e.g. CT scan, MRI) forced admission due to timeframes		• problems with transport
		• cost of medications or investigations
**Problems with outpatient services (10 admissions coded to this group):**		**Poor physical and/or cognitive functioning (20 admissions coded to this group):**
• access to outpatient angiography/cardiac diagnostics		
		• mental health problems
		• poor cognitive function
• dialysis/other outpatient services not available on weekends		• poor mobility or physical functional status
**Ideas for General Practice (7 admissions coded to this group):**		
• having chronic care nursing at GP practice		
• having GP service available out of hours/weekends		

**Table 4:** Metropolitan district—coding of free-text comments for each of the 65 preventable admissions.

SYSTEMS Codes	CLINICIAN Codes	PATIENT codes
**Community based services missing or inadequate or not referred to (65 admissions coded to this group):**	**GP care inadequate (59 admissions coded to this group):**	**Problems with adherence/self-management (57 admissions coded to this group):**
	• inadequate GP medical management of existing chronic condition	
		• patient/carer requires education
		• inadequate self-management skills
community nursing, allied health, care coordination, integrated care program, Respiratory Ambulatory Care Service, rehabilitation, Post-Acute Care team (daily respiratory monitoring following discharge), Chronic and Complex Care (for those at high risk of readmission), Chronic Disease Management Program, social and welfare support, respite, drug & alcohol support, depression & anxiety management, palliative care, hospital in the home, Aged Care assessment/needs assessment, palliative care, respite, aged care		• inadequate self-management lifestyle factors e.g. smoking
	• lack of action plan	
		• poor adherence to medication
	• inadequate GP medical management of known conditions predisposing to admission e.g. mental health, co-morbidities	• poor adherence to other aspects of disease management e.g. fluid restriction
	• missing or inadequate GP Management Plan/Team Care Arrangements (Chronic Disease Management Plan)	
**Problems with specialist services (54 admissions coded to this group):**	**Both GP and hospital (20 admissions coded to this group):**	**Problems with patient’s engagement with existing services (45 admissions coded to this group):**
• Rapid Access and Stabilisation Service	• failure to refer to existing community based health service e.g. cardiac nursing	
• needing access to or referral to specialist services		• should have seen the GP earlier/sooner/more frequently
		• declined services
• unable to see specialist when required	• required home medicine review	• has a GP but insufficient or inadequate connection with GP
• needing to see specialist more often		
		• should have gone to GP not the hospital
**Other (33 admissions coded to this group):**	**Hospital care (8 admissions coded to this group):**	**Cost/logistics barriers to accessing services (23 admissions coded to this group):**
• could have been seen as outpatient or in the community (inappropriate admission)		
	• inadequate hospital management of existing condition	
		• physical/logistics barriers e.g. transport
	• specialist admitting unnecessarily for diagnostics that could have been done in the community	
		• cost barriers e.g. medicines, GP, specialist
**Poor communication and linkages between services (21 admissions coded to this group):**		**Poor functioning (13 admissions coded to this group):**
		• mental health problems
• between care providers		• poor cognitive function
• poor discharge practices (including discharged too early)		• poor mobility or physical functional status
**Ideas for General Practice (11 admissions coded to this group):**		**Support needed (10 admissions coded to this group):**
• having chronic care nursing at GP practice		• lack of social support
		• support from existing carer inadequate/not coping at home
• having GP service available out of hours/weekends		
		• support needed for carer/carer illness/death, and/or respite required
**Problems with outpatient services (3 admissions coded to this group):**		
• access to outpatient angiography/cardiac diagnostics		
• dialysis/other outpatient services not available on weekends		

#### Rural district

Table 3 (rural district) shows the considerable variation in the frequency with which each contributing factor group was used. The most commonly used groups were ‘Systems issues: Community based services missing or inadequate or not referred to’ which was coded for 79 of the 83 admissions; ‘Patient issues: Problems with adherence/self-management’ which was coded for 66 of the admissions; and ‘Clinician issues: GP care inadequate’ which was coded for 62 of the 83 admissions. Other groups used for more than 30 admissions were ‘Patient issues: problems with patient’s engagement with existing services’ (54 admissions); ‘Systems issues: Poor communication and linkages between services’ (43 admissions); ‘Systems issues: Problems with specialist services’ (35 admissions); ‘Patient issues: Support needed’ (34 admissions); and ‘Patient issues: Cost or logistical barriers to accessing services’ (30 admissions).

There was interaction between some of these issues. For example, 20 of the 35 admissions with ‘Systems issues: problems with specialist services’ were also coded to the group ‘Patient issues: Cost or logistical barriers to accessing services’.

‘Patient issues: Problems with adherence/self-management’ and ‘Patient issues: Problems with the patient’s engagement with existing services’ were the most frequent, or the only groups, identified for 14 of the 83 preventable admissions, indicating that for these admissions, patient behaviours were the main contributing factors. More detailed journal notes record that the detail in these groups included patients not adhering to medication and/or diet or fluid restrictions, patients not recognising symptoms and seeking help (actioning an action plan), and not seeing the GP frequently or regularly enough. In some cases there was evidence to suggest that these behaviours might be difficult to modify for these particular patients because of patient attitudes. For example, a patient with known CHF, diabetes, COPD and renal disease who had declined referral to a cardiac nurse for additional education, did not attend visits with the GP as frequently as requested by the GP, was non-compliant with salt restrictions and was admitted with acute pulmonary oedema, which responded well to intravenous frusemide.

#### Metropolitan district

The metropolitan district also had considerable variation in the frequency with which each issue was identified (Table 4). The most commonly used contributing factor group was ‘Systems issues: Community based services missing or inadequate or not referred to’. All 65 preventable admissions were coded to this group. The group contained a large number of codes (19 codes), the most commonly used of which were: *Missing or inadequate or not referred to allied health services including community nursing*; and *Missing or inadequate or not referred to care coordinator/care facilitator/case manager/integrated care program/GP liaison nurse*.

In the Clinician category ‘Clinician issues: GP care inadequate’ was the most commonly used group, coded for 59 of the 65 preventable admissions. This was largely driven by two issues: *inadequate GP management of existing chronic* condition (detailed journal notes record this was primarily medication management, and the need for closer monitoring/follow up/recall systems) and *lack of action plan*.

In the patient contributing factor groups (green), ‘Patient issues: Problems with adherence/self-management’ was the most commonly used group, used for 57 of the 65 admissions. This was primarily driven by the *Inadequate self-management skills* and *Patient requires education* codes.

#### Comparing rural and metropolitan district findings

**Fig 4:**
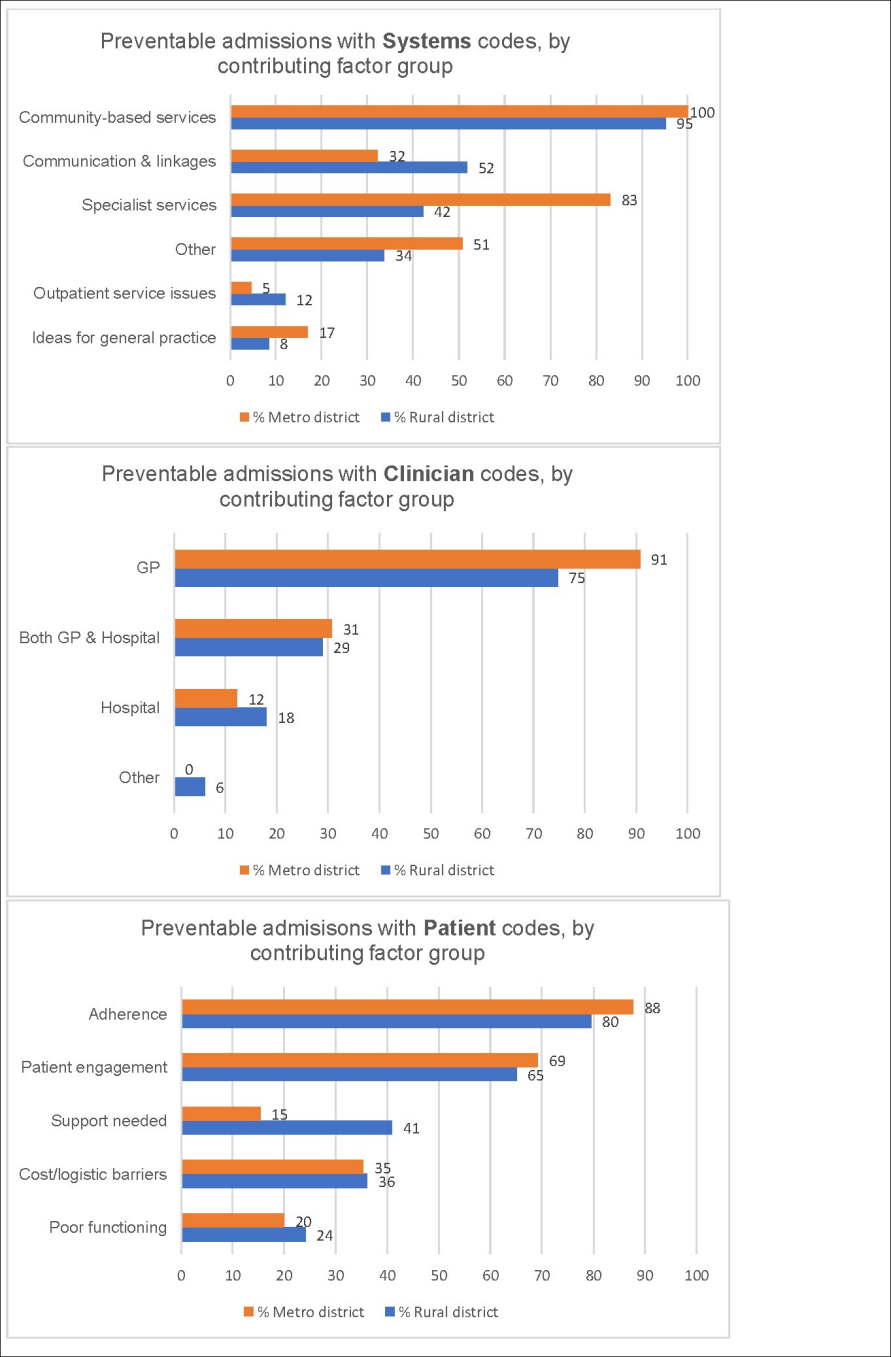
Coding of DaPPHne free-text comments. Preventable admissions coded to Systems, Clinician and Patient categories by contributing factor group (percentage of preventable admissions coded to each group).

Fig 4 shows coding of the free-text data within Systems, Clinician and Patient categories, by contributing factor group e.g. ‘Systems issues: Community based services missing or inadequate or not referred to’ (abbreviated to ‘Community based services’) comparing the rural and metropolitan districts.

Fig 4 illustrates the considerable variation in the frequency with which each contributing factor group was utilised.

There were similarities and differences between districts in the ranking of contributing factor groups overall and in the drivers of each group. All three categories: Systems, Clinician and Patient featured prominently. The top contributing factor group was the same in both districts, although the second ranking group differed (‘Patient: Problems with adherence/self-management’ in rural and ‘Clinician: GP care inadequate’ in metropolitan) (Table 5).

**Table 5:** Ranking of top 10 most commonly coded groups (across all three categories: System, clinician and patient) comparison between rural and metropolitan districts.

Rank Rural (across all 3 categories)	Contributing factor Group (proportion of preventable admissions coded to this group)	Rank Metro (across all 3 categories)	Contributing factor Group (proportion of preventable admissions coded to this group)
**1**	**Systems: Community based services missing or inadequate or not referred to (95%)**. Largely driven by: *Social and welfare support; Allied health services (including diabetes educator in particular)*	**1**	**Systems: Community based services missing or inadequate or not referred to (100%)**. Largely driven by: *Allied health services (including cardiac nursing in particular); Care coordinator/care facilitator/case manager/integrated care program/GP liaison nurse/connecting care program*
**2**	**Patient: Problems with adherence/self-management (80%).** Largely driven by: *Poor adherence to medication regimen; Patient requires education; Inadequate self-management skills*	**2**	**Clinician: GP care inadequate (91%).** Largely driven by: *Inadequate GP* management of *existing chronic condition (including medication management*, *closer monitoring/follow up/recall systems); Lack of action plan*
**3**	**Clinician: GP care inadequate (75%).** Largely driven by: *Inadequate GP management of existing chronic condition (including medication management*, *closer monitoring/follow up/recall systems); Lack of action plan*	**3**	**Patient: Problems with adherence/self-management (88%).** Largely driven by: *Inadequate self-management skills; Patient requires education; Inadequate self-management lifestyle factors e*.*g*. *smoking*
**4**	**Patient: Problems with patient’s engagement with existing services (65%).** Largely driven by: *Should have seen the GP earlier; Declined services; Should have seen the GP more frequently/regularly*	**4**	**Systems: Problems with specialist services (83%).** Largely driven by: *Rapid access and stabilisation service; Needing access to or referral to specialist services*
**5**	**Systems: Poor communication and linkages between services (52%).** Largely driven by: *Poor communication between providers; Poor discharge practices*	**5**	**Patient: Problems with patient’s engagement with existing services (69%).** Largely driven by: *Should have seen the GP earlier; Should have seen the GP more frequently/regularly*
**6**	**Systems: Problems with specialist services (42%).** Largely driven by: *Unable to see specialist when required; Needing access to specialist locally*	**6**	**Systems: Other (51%).** Largely driven by: *Could have been seen as outpatient or in the community*
**7**	**Patient: Support needed (41%).** Largely driven by: *Lack of social support*	**7**	**Patient: Cost/logistics barriers to accessing services (35%).** Largely driven by: *Physical/logistics barriers e*.*g*. *transport; Cost barriers*
**8**	**Patient: Cost/logistics barriers to accessing services (36%).** Largely driven by: *Physical/logistics barriers e*.*g*. *transport; Cost barriers*	**8**	**Systems: Poor communication and linkages between services (32%).** Largely driven by: *Poor communication between providers; Poor discharge practices*
**9**	**Systems: Other (34%).** Largely driven by: *Could have been seen as outpatient or in the community*	**9**	**Clinician: Both GP and hospital (31%).** Largely driven by: *failure to refer to existing community based health service e*.*g*. *cardiac nursing; Required home medicine review*
**10**	**Clinician: Both GP and hospital (29%).** Largely driven by: *failure to refer to existing community based health service e*.*g*. *palliative care*, *cardiac nursing; Required home medicine review*	**10**	**Patient: Poor functioning (20%).** Largely driven by:
			*Mental health problems*

Table 5 illustrates that the most commonly used contributing factor group in both districts was ‘Systems: Community based services missing or inadequate or not referred to’. However, the drivers differed; in the rural district social and welfare support was key whereas this was not a key driver in the metropolitan district. In both districts allied health ‘missing or inadequate or not referred to’ was a key driver in this group. In the metropolitan district this group was also driven by chronic disease programs ‘missing or inadequate or not referred to’ such as care coordination and integrated care (within and between acute and primary care) programs.

In the rural district, ‘Patient: Problems with adherence and self-management’ ranked second and was driven by medication adherence problems, patient requiring education and patient had inadequate self-management whereas in the metropolitan district ‘Clinician: GP care inadequate’ (driven by inadequate GP management and lack of action plans) ranked second. This contributing factor group was ranked third in the rural district and was driven by the same codes as in the metropolitan district. For the metropolitan district ‘Patient: Problems with adherence and self-management’ ranked third and had slightly different drivers from the rural district. The ranking of contributing factor groups then differs between the two districts.

Analyses of the coding by each of the four DaPPHne chronic conditions were undertaken. This work did not identify any differences in the general patterns described above.

### Complexity of admissions classed as preventable

**Fig 5:**
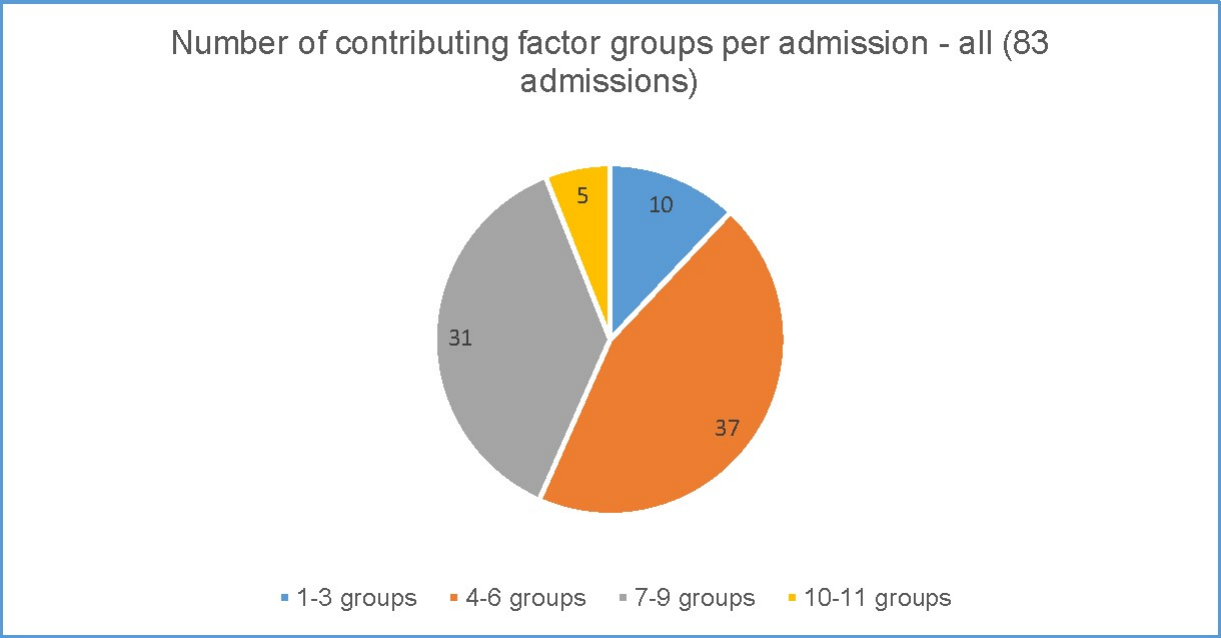
Complexity of preventable admissions–rural data.

**Fig 6:**
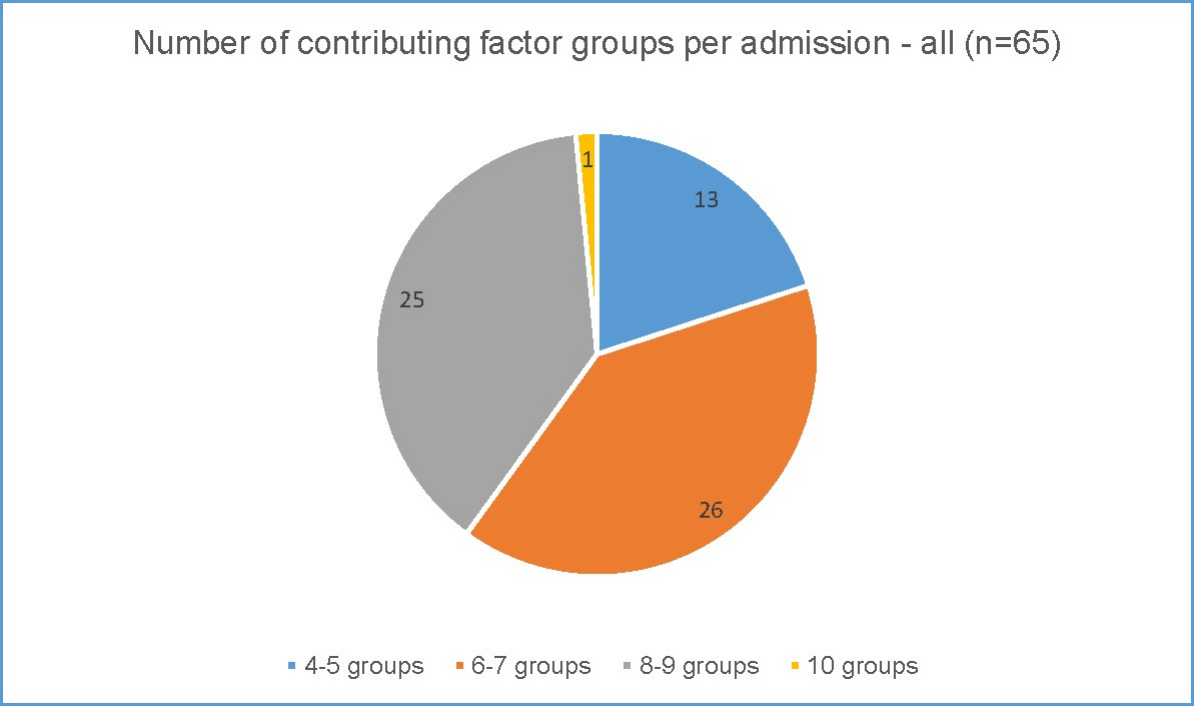
Complexity of preventable admissions–metropolitan data.

Figs 5 and 6 show the number of contributing factor groups used for each admission for the rural preventable admissions (Fig 5) and the metropolitan preventable admissions (Fig 6).

In the rural district (Fig 5), the number of groups coded for each admission varied from only one group to 11 (of the potential 15) groups. Larger numbers of contributing factor groups used indicates greater complexity in addressing the factors contributing to the admissions. Forty three percent (36 of the 83 preventable admissions) of all admissions were coded to a minimum of seven separate groups illustrating the range of factors that clinicians considered to have contributed to the preventable admission in the 3 months leading up to that admission. Even low numbers of groups i.e. only 2 or 3 contributing factor groups used, were usually from different categories (system, clinician and patient) showing multiple categories at play. Across all 83 preventable admissions in the rural district 72% were coded to contributing factor groups from each of the three categories. The number of groups coded also varied by principal discharge diagnosis, with COPD showing greater complexity.

In the metropolitan district (Fig 6), the number of groups coded for each admission varied from four groups to 10 groups from a possible 14. CHF showed greater complexity than the other conditions. Most of the admissions (51 of the 65 preventable admissions) were coded to 6–9 contributing factor groups illustrating the range of factors contributing to admissions deemed preventable. As with the rural district, even low numbers of groups i.e. only 4 or 5 contributing factor groups used, were usually from different categories (system, clinician and patient) showing multiple categories at play. Across all 65 preventable admissions in the metropolitan district 86% were coded to contributing factor groups from each of the three categories.

## Discussion

Our key findings from these analyses include that preventability is context specific. What clinicians considered to have contributed to PPH assessed as preventable and may have helped prevent that admission, whilst sharing broad similarities also reveals important differences between the rural and metropolitan districts. Furthermore, the Panels’ assessments showed different proportions of preventability by health district. As such, variations in rates of PPH (which are linked to health service funding) might not reflect variation in actually preventable hospitalisations and each district should review its PPH rate and assess preventability. Comparing rates and types of PPH as well as what might be done to prevent PPH between districts might not be productive. This approach is further supported by multivariate analyses of the DaPPHne data which included ‘site’ as a variable and demonstrated that ‘site’ was highly significant [8]. This illustrates that the proportion of PPH deemed preventable and what is associated with preventability is specific to each district.

The most commonly used contributing factor group for both districts was ‘Systems issues: Community based services missing or inadequate or not referred to’. In the rural district 95% were coded to this group and in the metropolitan district every preventable admission was coded to this group. This was driven by unmet social and welfare support needs and access to allied health services in the rural district, while in the metropolitan district it was driven by access to allied health services and various integrated care programs providing care coordination and case management. In the wider DaPPHne study, important predictors of admissions being classified as preventable were patients who needed help with everyday living tasks, and those who lived alone (Fig 2). Social and welfare support needs impact in multiple ways on patients’ capacity to manage their chronic condition [18]. These include opportunities that are offered by social interaction such as: facilitating access to services; improving pain tolerance, mental health and nutritional status and peer pressure around healthy behaviours [16] and are identified in the chronic disease and hospital avoidance literature [19, 20]. Clinicians in the rural district agreed that improvement in this support would have contributed to preventing some of the preventable admissions.

Lack of access (including affordability and long waits) to allied health services is well rehearsed in the literature, particularly in rural areas which face considerable challenges of recruitment and retention of the allied health workforce [21, 22]. The DaPPHne data confirm that clinicians view improvement in access to allied health services as having the potential to make a difference in the lead-up to a preventable admission. Indeed, chronic disease management programs aiming to reduce acute care usage commonly have an allied health dimension [23]. As researchers we were particularly struck by the plethora of programs available for patients with chronic conditions in the metropolitan district, each program with a slightly different aim and eligibility. Some aspects of ‘not referred to’ may be explained by clinicians struggling to keep abreast of the many different, and changing options available for this patient group. Improving access to these programs, partly by supporting clinicians in understanding and making referrals to them or by simplifying eligibility requirements, may contribute to preventing preventable hospital admissions amongst this patient group.

The second (rural) and third (metropolitan) most commonly used contributing factor group was ‘Patient: Problems with adherence/self-management’. In the rural district this was largely driven by the patient’s poor adherence to their medication regimen; the patient requiring education, and inadequate self-management skills. In the metropolitan district, it was driven by inadequate self-management skills; the patient requiring education, and inadequate self-management of lifestyle factors, for example smoking. These issues of self-management, often demanding behaviour change, are commonly identified in the PPH literature [24–26] and reflect the findings of another sub-study of the DaPPHne study which explored patient perspectives in greater depth [17]. Changing behaviours, many of which may have developed over a considerable time can be difficult and requires significant and sustained support.

The ‘Clinician issues: GP care inadequate’ group was the third (rural) and second (metropolitan) most commonly used contributing factor group. In both districts this group was largely driven by: inadequate GP management of the patient’s existing chronic condition (including medication management, closer monitoring/follow up/recall systems); and lack of action plans. In the rural district this issue is also reflected in the fourth most commonly used group (‘Patient issues: Problems with patients’ engagement with existing services’) largely driven by patients not seeing the GP early enough or needing to see the GP more frequently or regularly. This issue may also be related to continuity of care, where patients with ambulatory care sensitive conditions (including the four DaPPHne conditions) who see the same GP over time are less likely to have a PPH [27]. The effective use of action plans, where patients and clinicians access, understand and use a single plan, has been associated with decreased likelihood of ED presentation and hospital admission for COPD [28, 29]. A systematic review demonstrated the association between fewer admissions (all-causes and for CHF) and interventions that included aspects of action planning such as patients monitoring signs and symptoms and seeking care early if deterioration was apparent [30].

In the metropolitan site, ‘Systems issues: Problems with specialist services’, driven by lack of referral to the Rapid Access and Stabilisation Service (RASS) and needing access to specialists was a more commonly described suggestion (ranked fourth) for what might have been done in the three months leading up to the admission than in the rural district (ranked sixth). The RASS is for patients with a deteriorating health condition who are at risk of an admission (referred from the GP), or following discharge from hospital who are well enough to go home but require ongoing specialist review for up to three visits over five to seven days to ensure no relapse and readmission. The goal is for brief focused specialist care and transfer back to the GP for ongoing care. Given programs like RASS exist in the metropolitan district, precisely to address issues of preventable hospitalisation, there seems to be an issue with accessing it that would benefit from further investigation. This may account for the more frequent coding to ‘access to specialist services’ in the admissions from the metropolitan district compared to the rural district despite well documented limitations to specialist access in rural settings [31].

Our findings clearly illustrate that due to the complexity of factors contributing to the admissions assessed as preventable, there are no ‘magic bullets’ in terms of interventions to reduce PPH. There are, however, important lessons. Preventing individual preventable admissions will require an approach addressing a combination of systems, clinician and patient factors. Along with the general difficulty of identifying ‘impactible’ patients [32], this may explain the apparent failure to reduce admissions of many programs targeting complex long-term conditions [33–37]. This finding is supported by a systematic review of trials of interventions to reduce readmission which found greater effectiveness in multiple-component interventions [38]. In both districts most of the admissions had coding in all three of the main categories (system, clinician and patient). Even the simpler preventable admissions (those only coded to a small number of contributing factor groups) usually had a mixture of codes groups from all three categories (systems, clinician and patient).

Systems thinking and approaches may provide an important platform to explore and embrace the complexity of PPH in that the problems that drive the development of interventions are part of a complex movable and interconnecting system where a change in one part can affect another part [39].

Our understanding of which interventions work to reduce PPH is evolving over time and many interventions, whilst demonstrating improvements in quality of life measures and patient satisfaction do not report improvements in hospital admissions [6, 33, 34, 40, 41]. Indeed some studies have reported interventions which have led to increased admissions [6, 42]. As described earlier, this may be due to the context-specific nature of PPH, for example Hodgson et al conclude that:

*“…in many cases*, *the efficacy of an approach may be specific to particular healthcare contexts” p*.*431* [35]

Along with the findings from the rest of the DaPPHne study, the outputs of the analyses presented here were examined in face-to-face workshops (one for each of the two health districts). Findings were presented (and questions answered) and then discussed in small groups with the aim of contextualising the data, deepening understanding and supporting each health district to identify priorities for action. Overall understanding of the complexity of these admissions and what might be done about them developed amongst workshop participants. The following key areas for action in the rural district were identified: timely access to high quality GP care; patient self-management; establishing a locally driven navigation/coordination service; building a communication system to support service knowledge and understanding and clinical handover; increasing access to specialist and community services; and ensuring the system identifies and responds to patients who require additional support. In the metropolitan district agreed priorities were: targeting the right patients (including smarter use of systems to ‘flag’ patients who might be preventable); patient education and self-management; communication between services/clinicians; action planning, access to services/programs and medication management. There was also discussion of the DaPPHne findings that the admissions assessed as preventable were from a group of patients, most of whom had complex needs, with limited social and financial resources, multi-morbidity and associated polypharmacy. For many, their existing health conditions would be difficult to manage, highlighting the importance of prevention at a much earlier stage.

### Limitations of the study

Differences between admissions assessed and not assessed by the Expert Panels existed, including better health literacy, lower psychological distress and fewer diagnoses on admission among those assessed, indicating they may be slightly healthier than those not assessed by the Panel. Although the picture is quite complex this may mean that the admissions assessed were possibly slightly more likely to be assessed as preventable. In addition, inevitably, there were some limitations to the information available to the Panels. Whilst an enhancement of processes described in previous published work in this area [10, 11], the process still did not allow full insight into patient behaviours and circumstances.

## Conclusion

This study concludes that the factors contributing to the preventability of individual PPH are context specific and complex. This calls into question the use of PPH as a key performance indicator for both primary and secondary care. The use of PPH is clearly a ‘blunt’ instrument unable to account for differences in context and provides an overestimation of the proportion of PPH that are actually preventable. It is possible the measure could be refined with additional nuance which may improve its validity as a measure of preventable admission. Research to further understand PPH and develop interventions should be tailored to the specific context of each health district.

The complexity of factors contributing to admissions assessed as preventable underscores the importance of developing interventions which address systems, clinician and patient factors together (possibly adopting a systems thinking approach) in order to reduce PPH. In particular our findings illuminate the centrality of: community based services (including integrated care and case management services) to address unmet social, welfare and allied health support needs; support for patients’ self-management and adherence; and effective GP management of patients’ chronic condition/s including medication management, closer monitoring with systems for follow up and recall; and action plans.

## Supporting information

S1 File (DOCX)
