# Supplementary material for: What could prevent chronic condition admissions assessed as preventable in rural and metropolitan contexts? An analysis of clinicians’ perspectives from the DaPPHne study
Source: PLoS One. 2021 Jan 7;16(1):e0244313. doi: 10.1371/journal.pone.0244313 (PMC7790391; doi:10.1371/journal.pone.0244313)
Supplement: S1 File — (DOCX) [file pone.0244313.s001.docx]

*What could prevent chronic condition admissions assessed as preventable in rural and metropolitan contexts? An analysis of clinicians’ perspectives from the DaPPHne study*

Supplementary File – significant differences between admissions assessed by the panels and those not assessed:

A comparison of the 323 admissions assessed by the Expert Panels with the 222 admissions not assessed, identified differences between the groups. Those assessed by the Expert Panels were:

- more likely to have very good health literacy on the Partners in Health Scale(1) (57% vs 46%, p=0.009) and 9th grade or above scores on the REALM-R health literacy assessment tool(2) (73% vs 58%, p<0.001);
- less likely to have moderate or severe psychological distress on the K10 instrument(3) (34% vs 44%, p=0.022);
- less likely to have consulted their GP about the condition for which they were admitted to hospital, within the past three months (78% of those assessed vs 86% of those not, p=0.016);
- more likely to have a note in their hospital records that they were having difficulty managing at home (20% vs 9%, p<0.001);

There were no differences on final diagnoses (principal or total number), use of medications, or other sociodemographic characteristics. Given the lower rate of multi-morbidity, better health literacy and lower psychological distress among those assessed, they may be slightly healthier than those not assessed by the panel which may have resulted in an over-estimation of the proportion preventable.

*Supplementary Table: Demographic characteristics of admissions assessed for preventability compared to those not assessed*

|  | **Assessed**  **N=323**  **n (%)** | **Not assessed**  **N=222**  **n (%)** | **P-value** |
| --- | --- | --- | --- |
| ***Demographics*** |  |  |  |
| **Gender** |  |  | 0.624 |
| Male | 175 (54) | 125 (56) |  |
| Female | 148 (46) | 97 (44) |  |
| **Age** |  |  | 0.995 |
| 46-60 years | 57 (18) | 38 (17) |  |
| 60-70 years | 90 (28) | 64 (29) |  |
| 70-80 years | 109 (34) | 74 (33) |  |
| 80 and above | 67 (21) | 46 (21) |  |
| **Country of Birth** |  |  | 0.644 |
| Other countries | 93 (29) | 68 (31) |  |
| Australia | 230 (71) | 154 (69) |  |
| **Aboriginal/Torres Strait Islander** |  |  | 0.481 |
| Indigenous | 14 (4) | 7 (3) |  |
| Non-Indigenous | 309 (96) | 215 (97) |  |
| **Relationship Status** |  |  | 0.983 |
| Widowed/Divorced/Single | 164 (51) | 112 (51) |  |
| Married/De facto | 159 (49) | 109 (49) |  |
| **Living Alone** |  |  | 0.177 |
| Lives Alone | 104 (32) | 58 (27) |  |
| Other | 218 (68) | 158 (73) |  |
| **Highest Level of Education** |  |  | 0.376 |
| No School certificate or equivalent | 111 (35) | 85 (38) |  |
| School certificate or above | 210 (65) | 137 (62) |  |
| **Employment Status** |  |  | 0.435 |
| Retired | 240 (74) | 163 (74) |  |
| Employed | 27 (8) | 25 (11) |  |
| Others | 56 (17) | 33 (15) |  |
| **Household Income (AUD)** |  |  | 0.341 |
| <$20,000 | 105 (38) | 44 (31) |  |
| $20,000-$40,000 | 125 (46) | 70 (50) |  |
| >$40,000 | 44 (16) | 27 (19) |  |
| **Insurance Status** |  |  | 0.203 |
| Others | 244 (24) | 178 (80) |  |
| Private insurance | 79 (76) | 44 (20) |  |
| ***Self-reported health functioning*** |  |  |  |
| **Requires Daily Help** |  |  | 0.071 |
| Yes | 123 (38) | 102 (46) |  |
| No | 199 (62) | 120 (54) |  |
| ***Health literacy*** |  |  |  |
| **Partners in Health scale** |  |  | **0.009** |
| Very good | 185 (57) | 102 (46) |  |
| Satisfactory/very poor | 138 (43) | 119 (54) |  |
| **REALM score** |  |  | **<0.001** |
| 9^th^ grade or higher | 235 (73) | 126 (58) |  |
| Below 9^th^ grade | 87 (27) | 93 (43) |  |
| ***Self-reported social isolation, psychological distress*** |  |  |  |
| **Social support (Dukes)** |  |  | 0.712 |
| Little/No social support | 66 (21) | 48 (22) |  |
| Moderate/High social support | 250 (79) | 168 (78) |  |
| **Psychological distress - K10** |  |  | **0.022** |
| Moderate/High psychological distress | 109 (34) | 96 (44) |  |
| Well/Mild psychological distress | 211 (66) | 123 (56) |  |
| ***Use of prescription medications*** |  |  |  |
| **Total medications on admission** |  |  | 0.714 |
| Five and above | 254 (79) | 174 (80) |  |
| None to four | 68 (21) | 43 (20) |  |
| **Someone helps with medications** |  |  | 0.683 |
| Yes | 54 (17) | 40 (18) |  |
| No | 264 (83) | 178 (81) |  |
| ***Use of community-based services*** |  |  |  |
| **Saw GP about admission condition in previous 3 months** |  |  | **0.016** |
| Yes | 251 (78) | 190 (86) |  |
| No | 72 (22) | 31 (14) |  |
| ***Diagnoses (as recorded in hospital records)*** |  |  |  |
| **Principal diagnosis on discharge** |  |  | 0.834 |
| CHF | 79 (24) | 51 (23) |  |
| COPD | 123 (38) | 93 (42) |  |
| Diabetes | 88 (27) | 58 (26) |  |
| Angina/ACS | 33 (10) | 20 (9) |  |
| **Total diagnoses on discharge** |  |  | 0.147 |
| 1-2 conditions | 21 (7) | 14 (6) |  |
| 3-6 conditions | 147 (46) | 83 (37) |  |
| 7 and above | 155 (48) | 125 (56) |  |
| ***Current hospital admission*** |  |  |  |
| **Indication in hospital notes that patient having difficulty managing at home** |  |  | **<0.001** |
| Yes | 64 (20) | 19 (9) |  |
| No | 256 (80) | 201 (91) |  |

**References**

1. Battersby MW, Ask A, Reece MM, Markwick MJ, Collins JP. The Partners in Health scale: The development and psychometric properties of a generic assessment scale for chronic condition self-management. Aust J Prim Health. 2003;9(3):41-52.

2. Bass PF, Wilson JF, Griffith CH. A shortened instrument for literacy screening. J Gen Intern Med. 2003;18(12):1036-8.

3. Kessler RC, Barker PR, Colpe LJ, Epstein JF, Gfroerer JC, Hiripi E, et al. Screening for Serious Mental Illness in the General Population. Arch Gen Psychiatry. 2003;60(2):184-9.
